# Editorial: Genetic resources and conservation strategies for neotropical plant biodiversity

**DOI:** 10.3389/fpls.2025.1629546

**Published:** 2025-06-27

**Authors:** Enéas Ricardo Konzen, Miklos Maximiliano Bajay, Jorge Carlos Berny Mier y Teran, Marcos Vinícius Bohrer Monteiro Siqueira

**Affiliations:** ^1^ Centro de Estudos Costeiros, Limnológicos e Marinhos (CECLIMAR), Universidade Federal do Rio Grande do Sul, Imbé, RS, Brazil; ^2^ Centro de Educação Superior da Região Sul (CERES), Universidade do Estado de Santa Catarina, Laguna, SC, Brazil; ^3^ World Coffee Research, Portland, OR, United States; ^4^ Universidade do Estado de Minas Gerais, Unidade Frutal, Frutal, MG, Brazil

**Keywords:** neotropical plants, genetic diversity, habitat fragmentation, breeding, conservation

**Figure 1:**
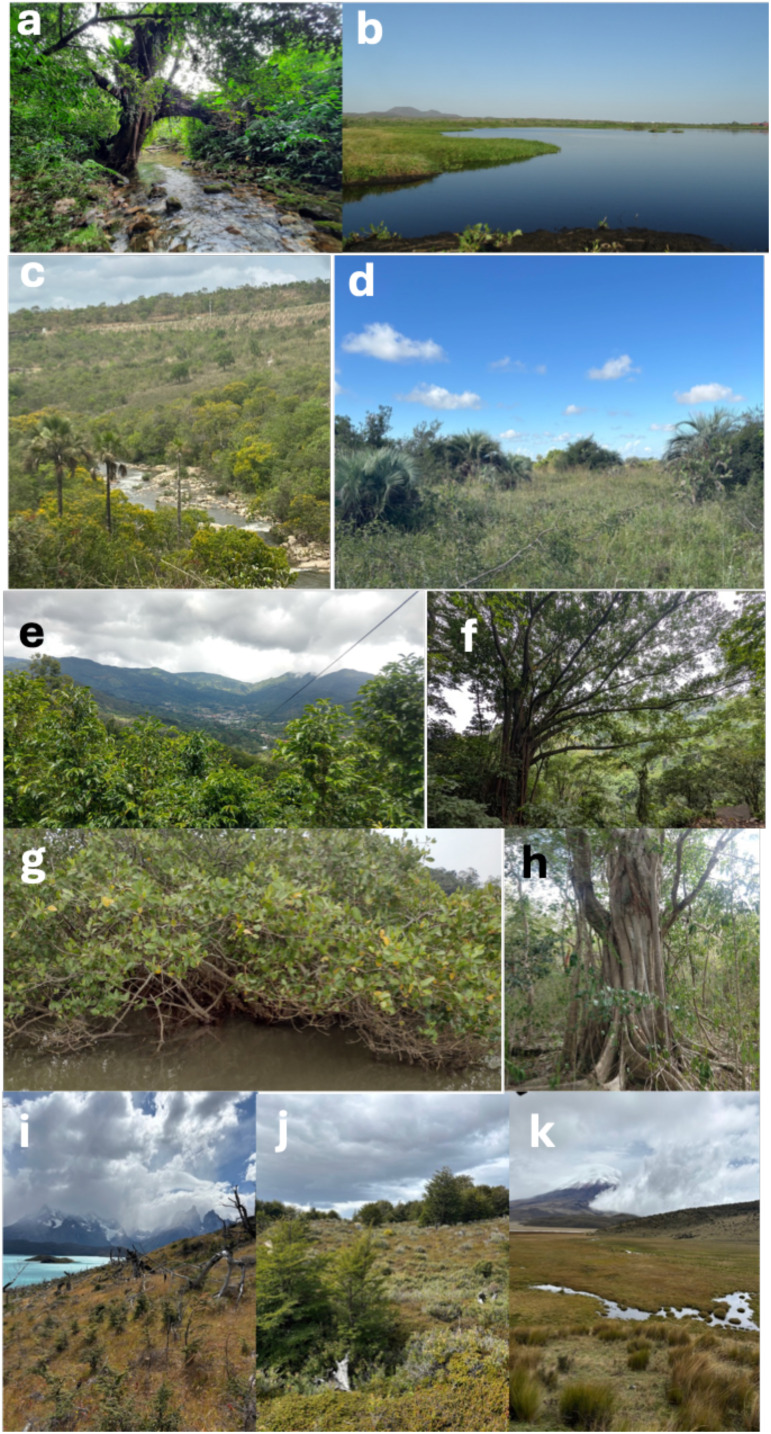
An overview of a few biomes and ecosystems that make up the megadiversity of plant species of the Neotropics. **(a)** Atlantic Rainforest, in Brazil. Credits: Marcos Siqueira. **(b)** Pantanal, in Brazil. Credits: Marcos Siqueira. **(c)** Cerrado (Brazilian savanna). Credits: Enéas Konzen. **(d)** Pampa. Credits: Enéas Konzen. **(e)** Coffee plantation next to a forest in Costa Rica. Credits: Jorge Berny. **(f)** El Salvador rainforest. Credits: Jorge Berny. **(g)** Mangroves in Southern Brazil, showing *Avicennia schaueriana*. Credits: Miklos Bajay. **(h)** *Ficus* sp. in a Yucatan´s medium subdeciduous forest. Credits: Jorge Berny. **(i)** Patagonia typical spot in Torres del Paine National Park, southern Chile. Credits: Enéas Konzen. **(j)** Reserva Magallanes, a Patagonian forest in southern Chile. Credits: Enéas Konzen. **(k)** Cotopaxi National Park, showing the Andean region of Ecuador and volcano Cotopaxi. Credits: Enéas Konzen.

The Neotropics are one of the most biodiverse biogeographic realms on the planet, with several plant species distributed from Central to South America (de Freitas and Galetti, 2025) (**Figure 1**). However, intense habitat fragmentation has compromised the survival of numerous species, even those of economic significance. A global meta-analysis on several organisms, including plants, already shows a reduction in the genetic diversity levels in neotropical species compared to all other realms, most likely due to human interference (Shaw et al., 2025).

In the neotropical region, there is insufficient knowledge regarding the potential applications of numerous endangered plant species for food security, pharmaceutical treatments, biodiesel, carbon stocks, among others. In comparison to other regions, neotropical plant genetic resources are still scarcely described (Bohrer Monteiro Siqueira et al., 2025), which demands further commitment from the scientific community to promote their conservation and breeding in times when climatic resilience is much needed. Recent studies have begun to explore the genetic diversity and structure of these plants using advanced molecular markers and sequencing technologies, but significant gaps remain in our understanding of their full potential and the best strategies for their conservation and utilization.

This Research Topic was aimed at acknowledging novel publications devoted to exploring the genetic resources, conservation, and breeding initiatives of researchers working with plant species belonging to the neotropical dominion, including trees, shrubs, herbs, and aquatic plants, endemic or widespread.

In one of the papers, Aguilar-Cano et al. invite you to explore the discovery of a hidden species from South America’s dry forests. The authors describe *Magoniella chersina*, a new liana species found in the seasonally dry tropical forests of northern Colombia and Venezuela. Overlooked for nearly a century, this plant was described through an integrative approach combining morphological and molecular data. The findings highlight the species distinct features, its isolated geographic range, and its likely endangered status. *M. chersina* stands out as the only representative of its genus in this dry biome, contrasting with its rainforest-dwelling relatives. Aguilar-Cano et al. demonstrate how field expeditions, herbarium research, and DNA sequencing can work together to shed light on overlooked biodiversity. This paper is an invitation to appreciate not only a new species, but also the importance of preserving the disappearing ecosystems where such discoveries are still possible.

Moreira-Muñoz et al. bring to us a compiled catalogue of the species of daisies (Asteraceae) family in South America. Their review showed a total of 6,940 species distributed in South America, placing the region as the most diverse for the Asteraceae family, accounting for approximately a quarter of the global diversity. The authors noted that the curves of species discovery have not yet stabilized, estimating that 5 to 10% more species can potentially be discovered. Among the countries involved in the catalogue, Brazil harbors the highest number of species (2,095), followed by Peru (1,588). The authors also acknowledged that the Chilean flora is consistently different from the other parts of the continent. Their manuscript also expressed a concern on the intense human interference with South American habitats, which challenges the conservation of the native daisies of the region.

Costa et al. investigated drought tolerance in cassava (*Manihot esculenta* Crantz), a primary starch-producing crop indigenous to the Neotropics. Its evolutionary history is marked by extensive clonal propagation and human-mediated selection, which have contributed to the vast phenotypic diversity observed in modern landraces. The study examined breeding strategies to enhance drought tolerance, which remains a major constraint to cassava productivity in semi-arid areas. Their findings indicate that genomic selection is an effective tool for breeding programs targeting resilience to water stress. By enabling the early prediction of genetic values for key agronomic traits associated with drought tolerance, genomic selection can significantly accelerate the development of improved varieties, thereby contributing to agricultural sustainability under limited irrigation scenarios.

Couto et al. explored genomic prediction and the optimization of training sets towards speeding up the improvement of the undomesticated perennial oil palm *Acrocomia aculeata*. They assessed different SNP calling methods, prediction models and optimization christeria, for three important traits: fruit and pulp dry mass and pulp oil content. The best predictions were made with the single nuclear polymorphism calling method using the oil palm as reference and Genomic best linear unbiased prediction as model. Training set optimization using percent explained variation, r-score and core collection provided the best approach. These approaches could be used in other undomesticated orphan crops.

The four articles illustrate a wide range of work that has been done toward conservation and breeding of neotropical plants. However, due to the megadiversity of species of this realm, much has yet to be described in the Neotropics. Moreover, several taxa of this realm already take part of the Earth BioGenome Project, an initiative to sequence the complete genomes of all known eukaryotic species (Lewin et al., 2018, 2022). Nevertheless, research groups that work *in situ* in the Neotropics are frequently underfunded and frequently need to seek for international networks and funding for moving forward with their research. Universities and other research institutes need more funding, as well as public policies that invest in qualified human resources for projects aimed at conservation and breeding of neotropical genetic resources, not only in the core research centers and universities, but also through widespread facilities throughout the distinct biomes that the Neotropics encompass. Researchers that live in the Neotropics should be the epicenters for research initiatives of their own biomes and species. Even this Research Topic could have more papers if all researchers in the field had support for open access publishing.
